# Does lack of resources impair access to breast and cervical cancer screening in Japan?

**DOI:** 10.1371/journal.pone.0180819

**Published:** 2017-07-13

**Authors:** Hiroshi Sano, Rei Goto, Chisato Hamashima

**Affiliations:** 1 Faculty of Economics, Shiga University, Hikone, Shiga, Japan; 2 Graduate School of Business Administration, Keio University, Yokohama, Kanagawa, Japan; 3 Division of Cancer Screening Assessment and Management, Center for Public Health Science, National Cancer Center, Chuo-ku, Tokyo, Japan; Leibniz Institute for Prvention Research and Epidemiology BIPS, GERMANY

## Abstract

**Objectives:**

To assess the impact of the quantity of resources for breast and cervical cancer screening on the participation rates in screening in clinical settings in municipalities, as well as to clarify whether lack of resources impairs access to cancer screening in Japan.

**Methods:**

Of the 1,746 municipalities in 2010, 1,443 (82.6%) and 1,469 (84.1%) were included in the analyses for breast and cervical cancer screening, respectively. In order to estimate the effects of the number of mammography units and of gynecologists on the participation rates in breast and cervical cancer screening in clinical settings, multiple regression analyses were performed using the interaction term for urban municipalities.

**Results:**

The average participation rate in screening in clinical settings was 6.01% for breast cancer, and was 8.93% for cervical cancer. The marginal effect of the number of mammography units per 1,000 women was significantly positive in urban municipalities (8.20 percent point). The marginal effect of the number of gynecologists per 1,000 women was significantly positive in all municipalities (2.54 percent point) and rural municipalities (3.68 percent point).

**Conclusions:**

Lack of mammography units in urban areas and of gynecologists particularly in rural areas impaired access to breast and cervical cancer screening. Strategies are required that quickly improve access for the residents and increase their participation rates in cancer screening.

## Introduction

The participation rates in breast and cervical cancer screening are lower in Japan than in Western countries. In 2010, the participation rates in breast and cervical cancer screening were 80.4% and 85.0%, respectively, in the United States, and 77.0% and 78.6%, respectively, in the United Kingdom, but only 36.4% and 37.7%, respectively, in Japan [1]. In a 2014 survey that asked approximately 1,800 Japanese people why they did not undergo cancer screening, the largest proportion (48.0%) indicated that they did not have time to go for screening [2]. The time and distance required to access cancer screening are also viewed as factors that lower the participation rates in other countries as well [3–6]. A systematic review of interventions to increase participation rates in cancer screening conducted by the United States Centers for Disease Control and Prevention (CDC) indicated that reducing the time and distance to access breast and colorectal cancer screening would be effective in increasing the participation rates [7,8].

In Japan, population-based screening for cancer was introduced in 1983, and municipalities are now responsible for conducting five kinds of cancer screening programs, including gastric, lung, colorectal, cervical, and breast cancers. The cancer screening programs in municipalities are implemented through mass surveys (mass screenings) that are done for people at designated times in medical facilities and screening vans (mobile screening units) and screening in clinical settings for individuals by appointment in medical facilities. Cancer screening in clinical settings was implemented to make it possible for people to participate in screening at a convenient time and in a desired nearby medical facility, in addition to the mass surveys [9]. However, the participation rate in screening in clinical settings among municipalities over the two-year period of 2012–2013 was only 18.8% and 25.5% for breast and cervical cancers, respectively [10].

In order to improve the availability of cancer screening in clinical settings in municipalities, sufficient resources for cancer screening need to be allocated in each municipality. However, in Japan, the number of gynecologists who conduct cervical cancer screening in clinical settings is low compared to Western countries. In Japan, the number of obstetricians and gynecologists per 100,000 women was approximately 15.6 in 2013, which was remarkably low compared to the OECD average of approximately 27.3 [11]. On the other hand, the number of mammography units relative to the Japanese female population was about the same as the number of units required for organized screening estimated using the data of the United Kingdom and the Netherlands, both of which have high participation rates in organized screening [12]. However, since this number included the number of mammography units used for opportunistic screening and for medical care services, it can be concluded that the number of mammography units for population-based screening in municipalities is insufficient.

Previous studies in the United States showed that people living in areas where the numbers of mammography units and primary care physicians were insufficient had low rates of breast and colon cancer screening [13–15]. In Japan, Takaku analyzed the relationship between the number of medical facilities, hospital beds, and public health nurses and the total participation rates in cancer screening in mass surveys and clinical settings in Japanese municipalities [9]. He found that the number of public health nurses affected the participation rates in gastric, lung, and colorectal cancer screening. However, public health nurses are mainly involved in mass surveys, so the impact of the quantity of medical resources on the participation rates in cancer screening in clinical settings was unclear.

The aim of this study was to assess the impact of the quantity of resources for breast and cervical cancer screening on the participation rates in clinical settings in municipalities, as well as to clarify whether lack of resources impairs access to breast and cervical cancer screening in Japan.

## Materials and methods

### Subjects

The subjects were selected from a total of 1,746 municipalities that conducted breast and cervical cancer screening in Japan. Participation rates in breast and cervical cancer screening in clinical settings were calculated for each municipality using the Report on Regional Public Health Services and Health Promotion Services (RRPHSHPS) between April 2010 and March 2011, which was prepared by the Ministry of Health, Labour and Welfare (MHLW). This report contained the number of persons eligible for cancer screening by sex and age and of participants in mass surveys and screening in clinical settings for all municipalities. Persons eligible for breast cancer screening conducted by municipalities included women aged ≥40 years, and those eligible for cervical cancer screening included women aged ≥20 years. In Basic Plan to Promote Cancer Control Programs since 2012, the MHLW has aimed to improve the participation rates in cancer screening of persons aged 69 and under [16]. Thus, the participation rates in breast and cervical cancer screening were calculated of women aged 40–69 years and 20–69 years, respectively.

Municipalities were excluded from the analyses if there were missing values in the variables used in this study. In addition, 38 and 21 municipalities that did not perform mammography and Pap smears for breast and cervical cancer screening, respectively, were also excluded. For breast and cervical cancers in Japan, mammography and Pap smears are recommended for population-based screening [17,18]. Furthermore, 46 municipalities in Iwate, Miyagi, and Fukushima Prefectures were excluded because they were affected by the Great East Japan Earthquake of March 2011. Of the 1,746 municipalities, 1,443 (82.6%) and 1,469 (84.1%) were included in the analyses for breast and cervical cancer screening, respectively.

### Quantity of resources

The quantity of resources for cancer screening in a municipality reflected the ease of access to cancer screening in the area. The numbers of mammography units and of gynecologists were used for the quantity of resources for breast and cervical cancer screening, respectively. The Data Book of Medical Devices and Systems between 2010 and 2011 contains lists of mammography units installed in all medical facilities in Japan as of October 1, 2009 [19]. From the number of mammography units by medical facility, the number of units by municipality was calculated. Mobile mammography vans owned by each facility were excluded from the calculation because they are mostly used in mass surveys for breast cancer. For the number of gynecologists in a municipality, the sum of the obstetrics/gynecology doctors and gynecologists in the Survey of Physicians, Dentists, and Pharmacists in 2010, conducted by the MHLW, was used. The numbers of mammography units and of gynecologists were calculated per 1,000 women in the population as of March 31, 2010, prepared by the Ministry of Internal Affairs and Communications (MIC).

### Covariates

Besides making screening accessible, the CDC also indicated that using client reminders and small media, including one-on-one education by telephone or interviews, and reducing out-of-pocket costs would be effective interventions to increase breast or cervical cancer screening rates [7,8,20]. It is known that these interventions can also contribute to improving the participation rates in cancer screening in Japan [21–26]. Proxy variables for these interventions that affect the participation rate were used in this study. Based on the data in the Survey regarding the Implementation of Cancer Screenings among Municipalities, conducted by the MHLW, similar strategies for client reminders, one-on-one education, and reducing out-of-pocket costs were selected for implementation in each of the municipalities as of January 2010: specifically, sending personal invitation letters, personal visits by community health workers, and free screening.

The characteristics of persons eligible for screening in each municipality were also used as covariates. The number of eligible persons aged 69 and under, and percentage of those aged 65–69 years for all municipalities were calculated using RRPHSHPS between April 2010 and March 2011.

Additionally, the characteristics of municipalities that affect the participation rates in cancer screening were also used in this study. According to previous studies in Japan, factors affecting the participation rates in cancer screening were occupation, place of employment, kind of public health insurance coverage [22,24,26–29], income level [9,22,28,29], health status of eligible persons [22,30], and financial condition of municipalities [9]. Since the kind of public health insurance coverage is determined by occupation and place of employment in Japan, the percentage of full-time employees among female workers in the municipality, not including the agriculture, forestry, and fishing industries, was calculated from the Population Census of Japan in 2010, conducted by the MIC. For the income level of the residents of municipalities, the annual income per person covered by National Health Insurance (the public health insurance run by each municipality) as of 2009 was obtained from the Survey on the Insured of National Health Insurance (NHI) in 2010, conducted by the MHLW. For the health status of the residents of municipalities, the percentage of those who were certified as requiring long-term care at Care Level 2 or greater in residents aged 40–74 years was obtained from the Report on Long-term Care Insurance in 2009. This was because the rate of residents certified at Care Level 2 or greater in long-term care system were used for estimating the healthy life expectancy of the residents of municipalities in Japan [31]. For the financial condition of the municipality, the financial capability indicator (the ratio of standard revenue to standard financial needs) and the ordinary balance ratio (ordinary expenses as a percentage of ordinary revenue) as of March 31, 2010 were obtained from the Report on Local Government Expenditures in Japan in 2009, conducted by the MIC. Furthermore, as a proxy variable for the ease of access to mass surveys in municipalities, the percentage of women who participated in mass surveys relative to all women who participated in screening in the previous year (2009) was used for breast and cervical cancers, respectively, obtained from RRPHSHPS between April 2009 and March 2010. Some municipalities may not make positive efforts to conduct cancer screening in clinical settings, instead of improving access to mass surveys for the residents by a large number of locations available for this or a greater number of examination days. The reason the number of participants in the previous year was used was to avoid simultaneous equation bias in the regression analysis [32].

### Statistical analysis

Multiple regression analyses were performed using the participation rates in breast and cervical cancer screening in clinical settings as dependent variables, and the strategies for improving the participation rates including the quantity of resources, the characteristics of eligible persons, and the characteristics of municipalities as independent variables. For all strategies other than the quantity of resources, a binary variable that takes the value 0 or 1was used to indicate whether they were implemented. The Tobit model was used for the estimate because the participation rates in screening ran from 0% through 100% [33]. Among municipalities, there were none for which the participation rates in breast cancer and cervical cancer screening in clinical settings were 100%, while the participation rates in screening in clinical settings was 0% for breast cancer in 338 municipalities and for cervical cancer in 202 municipalities. The relationships between the participation rate in breast and cervical cancer screening in clinical settings and the quantity of resources among municipalities were represented using a scatter diagram in S1 and S2 Figs of the Supporting information, respectively. The impact of each independent variable on the participation rate was evaluated as a marginal effect. In Tobit model, the change in a dependent variable induced by a one-unit change in each independent variable is measured by the marginal effect rather than the coefficient.

Two regression models were estimated in order to assess the differences in the impact of four strategies on the participation rates between urban and rural municipalities. Previous studies reported that the participation rates in cancer screening were different depending on whether the person eligible for screening lived in an urban area [3,28,34–36]. The interaction terms of four strategies and a binary variable to show whether the municipality was urban (a city or the 23 wards of Tokyo) were included in independent variables of Model 2, while the interaction terms were not included in those of Model 1. In Model 2, the marginal effects of the interaction terms indicated the additional impacts on the participation rates in urban municipalities (cities and wards) relative to rural municipalities (towns and villages). A binary variable to show whether the municipality was an ordinance-designated city (with a population of over 500,000) or the 23 wards of Tokyo and a binary variable to show whether the municipality was a village were also used in Model 1 and 2. All statistical analyses were performed using Stata 13.

## Results

The characteristics of breast cancer screening in clinical settings in Japan are presented in Table 1. The average participation rate in screening in clinical settings was 6.01%. The average number of mammography units per 1,000 women was 0.045 in all municipalities and 0.061 in urban. According to the average values of the other three strategies for improving the participation rates, sending personal invitation letters was implemented in about half of municipalities, while personal visits by community health workers and free screening were not implemented by even 10% of municipalities, whether urban or rural. Personal visits by community health workers in urban municipalities were implemented less than half the time in all municipalities.

**Table 1:** Characteristics of breast cancer screening in clinical settings in Japan in 2010.

Variable	Mean	S.D.	Min	Max
Number of municipalities	1443			
Number of urban municipalities	687 (47.6%)			
Participation rates in screening in clinical settings (%)	6.01	7.15	0.00	70.17
Strategies				
Number of mammography units per 1,000 women	0.045	0.060	0.000	0.486
Number of mammography units per 1,000 women in urban municipalities	0.061	0.041	0.000	0.262
Sending personal invitation letters ^b)^	0.544	0.498	0.000	1.000
Sending personal invitation letters in urban municipalities ^b)^	0.502	0.500	0.000	1.000
Personal visits by community health workers ^b)^	0.063	0.243	0.000	1.000
Personal visits by community health workers in urban municipalities ^b)^	0.029	0.168	0.000	1.000
Free screening ^b)^	0.029	0.168	0.000	1.000
Free screening in urban municipalities ^b)^	0.028	0.164	0.000	1.000
Characteristics of eligible persons				
Number of eligible persons (1,000 women)	8.6	19.4	0.0	355.3
Percentage of those aged 65–69 years	21.96	6.72	0.00	50.00
Characteristics of municipalities				
Percentage of women participating in mass surveys in 2009	72.31	32.05	0.00	100.00
Percentage of female full-time employees	37.51	5.71	9.88	56.54
Annual income per NHI insured (1,000 yen)	564.0	182.6	151.4	2107.2
Percentage of persons requiring long-term care	0.775	0.200	0.000	1.670
Ordinary balance ratio (%)	88.75	6.59	52.90	144.10
Financial capability indicator	0.57	0.33	0.05	2.77
Ordinance-designated city or 23 wards of Tokyo ^b)^	0.01	0.10	0.00	1.00
Village ^b)^	0.09	0.29	0.00	1.00

Note: S.D. = standard deviation;

^b)^ = binary variables; and NHI = National Health Insurance.

Table 2 shows the characteristics of cervical cancer screening in clinical settings in Japan. The average participation rate in screening in clinical settings was 8.93%. The average number of gynecologists per 1,000 women was 0.094 in all municipalities and 0.151 in urban. The implementation rate of the other three strategies for improving participation rates was similar to that of breast cancer screening.

**Table 2:** Characteristics of cervical cancer screening in clinical settings in Japan in 2010.

Variable	Mean	S.D.	Min	Max
Number of municipalities	1469			
Number of urban municipalities	696 (47.4%)			
Participation rates in screening in clinical settings (%)	8.93	9.14	0.00	57.98
Strategies				
Number of gynecologists per 1,000 women	0.094	0.144	0.000	1.638
Number of gynecologists per 1,000 women in urban municipalities	0.151	0.134	0.000	1.638
Sending personal invitation letters ^b)^	0.556	0.497	0.000	1.000
Sending personal invitation letters in urban municipalities ^b)^	0.519	0.500	0.000	1.000
Personal visits by community health workers ^b)^	0.063	0.242	0.000	1.000
Personal visits by community health workers in urban municipalities ^b)^	0.029	0.167	0.000	1.000
Free screening ^b)^	0.052	0.223	0.000	1.000
Free screening in urban municipalities ^b)^	0.065	0.246	0.000	1.000
Characteristics of eligible persons				
Number of eligible persons (1,000 women)	13.2	31.1	0.0	577.2
Percentage of those aged 65–69 years	15.64	5.90	0.00	40.45
Characteristics of municipalities				
Percentage of women participating in mass surveys in 2009	60.61	36.19	0.00	100.00
Percentage of female full-time employees	37.56	5.69	9.88	56.54
Annual income per NHI insured (1,000 yen)	563.2	184.1	151.4	2107.2
Percentage of persons requiring long-term care	0.775	0.204	0.000	1.670
Ordinary balance ratio (%)	88.72	6.60	52.90	144.10
Financial capability indicator	0.57	0.33	0.05	2.77
Ordinance-designated city or 23 wards of Tokyo ^b)^	0.01	0.11	0.00	1.00
Village ^b)^	0.09	0.29	0.00	1.00

Note: S.D. = standard deviation;

^b)^ = binary variables; and NHI = National Health Insurance.

The results for the determinants of the participation rates in breast cancer screening in clinical settings are presented in Table 3. The marginal effect of the number of mammography units per 1,000 women was not significant in Model 1, but that of the interaction term for urban municipalities was significantly positive in Model 2. For the other strategies for improving participation rates, personal visits by community health workers had a positive effect in both models, while the interaction term for urban municipalities was not significant in Model 2.

**Table 3:** Determinants of the participation rates in breast cancer screening in clinical settings in Japan.

	Model 1		Model 2	
Independent Variable	Coefficients	Marginal Effects	Coefficients	Marginal Effects
Strategies				
Number of mammography units per 1,000 women	0.886 (2.827)	0.714 (2.278)	-2.634 (3.184)	-2.124 (2.567)
Number of mammography units per 1,000 women × Urban municipality ^b)^	-	-	12.806 * (5.407)	10.324 * (4.359)
Sending personal invitation letters ^b)^	0.555 (0.327)	0.446 (0.263)	0.419 (0.425)	0.337 (0.342)
Sending personal invitation letters ^b)^ × Urban municipality ^b)^	-	-	0.236 (0.544)	0.191 (0.442)
Personal visits by community health workers ^b)^	1.576 * (0.675)	1.318 * (0.584)	2.097 ** (0.768)	1.774 ** (0.677)
Personal visits by community health workers ^b)^ × Urban municipality ^b)^	-	-	-2.260 (1.559)	-1.689 (1.065)
Free screening ^b)^	0.834 (0.913)	0.687 (0.768)	1.733 (1.230)	1.460 (1.076)
Free screening ^b)^ × Urban municipality ^b)^	-	-	-1.805 (1.818)	-1.372 (1.292)
Characteristics of eligible persons				
Number of eligible persons (1,000 women)	-0.055 *** (0.013)	-0.044 *** (0.010)	-0.062 *** (0.013)	-0.050 *** (0.011)
Percentage of those aged 65–69 years	0.134 *** (0.024)	0.108 *** (0.020)	0.130 *** (0.025)	0.105 *** (0.020)
Characteristics of municipalities				
Percentage of women participating in mass surveys in 2009	-0.194 *** (0.005)	-0.157 *** (0.005)	-0.191 *** (0.006)	-0.154 *** (0.005)
Percentage of female full-time employees	0.090 ** (0.033)	0.072 ** (0.027)	0.092 ** (0.033)	0.074 ** (0.027)
Annual income per NHI insured (1,000 yen)	0.003 * (0.001)	0.002 * (0.001)	0.003 * (0.001)	0.002 * (0.001)
Percentage of persons requiring long-term care	1.866 * (0.852)	1.503 * (0.687)	1.920 * (0.852)	1.548 * (0.687)
Ordinary balance ratio (%)	-0.054 * (0.026)	-0.043 * (0.021)	-0.065 * (0.027)	-0.052 * (0.021)
Financial capability indicator	0.413 (0.609)	0.333 (0.491)	0.265 (0.611)	0.214 (0.492)
Ordinance-designated city or 23 wards of Tokyo ^b)^	6.430 ** (2.174)	5.853 ** (2.118)	7.143 ** (2.189)	6.553 ** (2.147)
Village ^b)^	-1.260 * (0.615)	-0.982 * (0.462)	-1.261 * (0.621)	-0.984 * (0.467)
Constant	14.345 *** (2.902)	-	14.925 *** (2.950)	-
Number of municipalities	1443		1443	
Pseudo R2 and likelihood-ratio Chi2	0.1286	1099.20 ***	0.1298	1109.15 ***

Note:

*** = P values≦0.001;

** = P values≦0.01;

* = P values≦0.05;

Standard errors in parentheses; and b) = binary variables. The marginal effects describe the changes in the participation rate induced by a one-unit change in each independent variable.

With regard to the characteristics of persons eligible for breast cancer screening, in both Model 1and 2, the number of eligible persons had a negative effect, while the percentage of those aged 65–69 years had a positive effect. For the characteristics of municipalities, the percentage of female full-time employees, the annual income per NHI insured, the percentage of persons requiring long-term care and the binary variable for ordinance-designated cities had positive effects in both models. The percentage of women participating in mass surveys in the previous year, the ordinary balance ratio and the binary variable for villages had a negative effect in both models.

Table 4 provides the results for the determinants of the participation rates in cervical cancer screening in clinical settings. The marginal effect of the number of gynecologists per 1,000 women was significantly positive in both Model 1 and 2, while that of the interaction term for urban municipalities was not significant in Model 2. Sending personal invitation letters had a positive effect in both models, and the interaction term for urban municipalities also had a positive in Model 2. Personal visitations by community health workers had a positive effect in both models, while the interaction term for urban municipalities had no effect in Model 2.

**Table 4:** Determinants of the participation rates in cervical cancer screening in clinical settings in Japan.

	Model 1		Model 2	
Independent Variable	Coefficients	Marginal Effects	Coefficients	Marginal Effects
Strategies				
Number of gynecologists per 1,000 women	2.864 * (1.352)	2.543 * (1.200)	4.141 * (1.867)	3.679 * (1.659)
Number of gynecologists per 1,000 women × Urban municipality ^b)^	-	-	-3.200 (2.496)	-2.843 (2.218)
Sending personal invitation letters ^b)^	2.173 *** (0.379)	1.920 *** (0.334)	1.254 ** (0.480)	1.112 ** (0.424)
Sending personal invitation letters ^b)^ × Urban municipality ^b)^	-	-	1.827 ** (0.584)	1.645 ** (0.532)
Personal visits by community health workers ^b)^	2.096 ** (0.782)	1.909 ** (0.728)	2.191 * (0.888)	1.999 * (0.829)
Personal visits by community health workers ^b)^ × Urban municipality ^b)^	-	-	-1.148 (1.780)	-1.001 (1.520)
Free screening ^b)^	-0.226 (0.818)	-0.200 (0.721)	0.610 (1.245)	0.547 (1.124)
Free screening ^b)^ × Urban municipality ^b)^	-	-	-1.347 (1.617)	-1.171 (1.372)
Characteristics of eligible persons				
Number of eligible persons (1,000 women)	-0.046 *** (0.009)	-0.041 *** (0.008)	-0.047 *** (0.010)	-0.042 *** (0.009)
Percentage of those aged 65–69 years	0.290 *** (0.033)	0.257 *** (0.029)	0.289 *** (0.033)	0.256 *** (0.029)
Characteristics of municipalities				
Percentage of women participating in mass surveys in 2009	-0.201 *** (0.006)	-0.179 *** (0.006)	-0.200 *** (0.006)	-0.178 *** (0.006)
Percentage of female full-time employees	0.117 ** (0.038)	0.104 ** (0.034)	0.116 ** (0.038)	0.103 ** (0.034)
Annual income per NHI insured (1,000 yen)	0.001 (0.001)	0.001 (0.001)	0.001 (0.001)	0.001 (0.001)
Percentage of persons requiring long-term care	0.915 (0.970)	0.812 (0.861)	0.995 (0.968)	0.885 (0.860)
Ordinary balance ratio (%)	-0.110 *** (0.030)	-0.098 *** (0.027)	-0.122 *** (0.031)	-0.108 *** (0.027)
Financial capability indicator	3.500 *** (0.731)	3.108 ** (0.650)	3.419 *** (0.731)	3.038 *** (0.650)
Ordinance-designated city or 23 wards of Tokyo ^b)^	8.962 *** (2.488)	8.596 *** (2.471)	9.433 *** (2.505)	9.069 *** (2.491)
Village ^b)^	-0.462 (0.695)	-0.407 (0.610)	-0.193 (0.702)	-0.171 (0.621)
Constant	16.941 *** (3.346)	-	18.180 *** (3.410)	-
Number of municipalities	1469		1469	
Pseudo R2 and likelihood-ratio Chi2	0.1192	1179.98 ***	0.1203	1190.66 ***

Note:

*** = P values≦0.001;

** = P values≦0.01;

* = P values≦0.05;

Standard errors in parentheses; and b) = binary variables. The marginal effects describe the changes in the participation rate induced by a one-unit change in each independent variable.

The characteristics of persons eligible for cervical cancer screening showed similar trends to those for breast cancer screening. With regard to the characteristics of municipalities, the following variables were different from the result for breast cancer screening: the financial capability indicator had a positive effect in both Model 1 and 2, and the annual income per NHI insured, the percentage of persons requiring long-term care and the binary variable for villages had no effects in both models.

## Discussion

The extent to which various strategies can improve the participation rate in cancer screening has been investigated in developed countries, and the effectiveness depends on the country and on the area [4,21,37,38]. In Japan, the strategies implemented by municipalities, namely sending personal invitation letters [21,25,26], distribution of leaflets and pamphlets [24], reduction of copayments [22,23], and increasing the availability of screening in clinical settings [9], have been shown to be effective in improving the participation rate in cancer screening. However, the relationships between the quantity of medical resources and the participation rates in cancer screening in clinical settings for improving access to screening have not been clear. The present study assessed the impacts of the numbers of mammography units and gynecologists on participation rates in breast and cervical cancer screening, respectively, in clinical settings in municipalities, which clarified to what extent a lack of resources has impaired access to cancer screening in Japan.

An increase in the number of mammography units per 1,000 women increased the participation rates in breast cancer screening in clinical settings in urban municipalities, but it had no effect on the participation rates in rural municipalities. In Model 2, an increase of 1 mammography unit per 1,000 women increased the participation rate in screening by 8.20 percent point ((-2.12)+10.32) in urban municipalities. Conversely, if the number of mammography units per 1,000 women in a municipality were to decrease by 0.26 units (the difference between the maximum value and the minimum value in urban), a reduction of 2.15 percent point in the screening participation rate would be expected. Even in urban areas, the number of mammography units was small, and 11.4% (78/687) of all cities and wards did not have mammography units. In rural areas, 79.1% (598/756) of the towns and villages did not have mammography units, and the fact that a very large number of municipalities lacked mammography units is believed to be one reason why this did not affect the screening participation rate in Model 1 and in rural municipalities in Model 2.

An increase in number of gynecologists per 1,000 women increased the participation rates in cervical cancer screening in clinical settings in all municipalities, and had a particularly large effect on the participation rates in rural. In all municipalities, an increase of one gynecologist per 1,000 women can be expected to increase the screening participation rate by 2.54 percent point, and moreover to increase the participation rate by 3.68 percent point in rural. Conversely, if the number of gynecologists were to decrease by 1.64 per 1,000 women (the difference between the maximum value and the minimum value), a reduction of 4.17 percent point and 6.03 percent point in the screening participation rate would be predicted in all and rural municipalities, respectively. In Model 2, the number of gynecologists had less impact on the participation rate in urban municipalities, and it might be one reason why many gynecologists were not devoted to screening in clinical settings but to mass surveys in urban areas. The result for multiple regression analysis to examine the impact of the quantity of resources for breast and cervical cancer screening on the participation rate in mass surveys in 2010 was shown in S1 Table of the Supporting information. The independent variables were the same as in Tables 3 and 4, except that the percentage of women participating in mass surveys in the previous year was changed to the percentage of women participating in clinical settings in the previous year, and the Tobit model was used for the estimate. Increasing the number of gynecologists improved the participation rate in mass surveys in urban municipalities.

Previous studies showed that people who live a short distance from a screening facility were more likely to participate in cancer screening [3–5]. In addition, people who underwent screening by a primary care physician or a gynecologist during the previous 12 months and people who had many health care visits were also likely to participate in cancer screening [35,36,39]. Screening advice by health care providers including physicians also had the effect of increasing the participation rate in cancer screening [39,40]. These benefits can be realized by increasing the numbers of screening units and physicians for cancer screening in a given area. In Japan, the fact that the number of mammography units is low even in urban areas and the number of gynecologists is particularly low in rural areas obstructs access to breast and cervical cancer screening for the residents. Therefore, to increase the participation rates in breast cancer screening, more mammography units should be allocated in urban areas. With regard to cervical cancer screening, by increasing the number of gynecologists particularly in rural areas, we can expect to improve the availability of screening.

However, it is not easy to increase the resources of facilities to complete cancer screening in clinical settings by increasing the number of mammography units and gynecologists in these areas. Therefore, it may not be possible to implement such strategies quickly. In particular, in Japan it usually takes a minimum of 8 years for a student to complete medical school and general residency, after which they may choose a specialty such as gynecology [41]. The estimates showed, for all samples, that the percentage of women who participated in mass surveys in the previous year had a negative effect. In municipalities where it is easy to arrange mass surveys, which have a large number of locations available for this or a greater number of examination days, it is believed that residents will select mass surveys rather than screening in clinical settings. Access to breast cancer screening can also be improved by providing mobile mammography vans within municipalities [42,43]. It is important to try to promptly improve access for people, not just by increasing the number of mammography units installed in medical facilities, but also by increasing the number of locations and dates of mass surveys by using mobile mammography vans.

In order to quickly improve access to cervical cancer screening, primary care physicians other than gynecologists should also perform Pap smears. Gynecologists perform Pap smears in Japan, while primary care physicians mainly perform Pap smears in the United Kingdom [44]. In some European countries, midwives have performed Pap smears for cervical cancer screening. However, in Japan, allied medical personnel have been prohibited from performing medical practice including Pap smears, and primary physicians do not perform Pap smears. In Japan, the number of primary care physicians per population at clinics was 7.5 times the number of gynecologists per population at clinics in 2012. By training primary care physicians other than gynecologists to perform Pap smears, it is possible to increase the quantity of resources for cervical cancer screening. Having women collect the specimen by themselves for cervical cancer testing also makes it easy for women to access cervical cancer screening. An HPV test with a self-collected sample has about the same accuracy as when the sample is collected by the physician [45,46]. Previous studies showed that HPV tests with self-collected samples are effective in motivating women to be screened who were not undergoing cervical cancer screening in rural areas [47,48]. In municipalities where it is difficult to secure gynecologists, these strategies will improve the cancer screening participation rate.

With regard to the other strategies for improving participation rates in both breast and cervical cancer screening, personal visits by community health workers increased the participation rates in screening in clinical settings. Personal visits by community health workers increased the participation rate in breast cancer screening by 1.32 percent point and 1.77 percent point in all and rural municipalities, respectively, and in cervical cancer screening by 1.91 percent point and 2.00 percent point in all and rural municipalities, respectively. However, these are also low compared to the effect of one-on-one education in increasing the participation rates in other countries [20]. Community health workers are municipal officials who are licensed as a public health nurse or nutritionist, and citizen volunteers who have received the training about preventive measures for cancers. There were many municipalities where personal visits were performed by the residents receiving the training rather than by municipal officials [50], and personal visits by community health workers who had little expertise might lower the effect of this strategy. For cervical cancer screening, sending personal invitation letters also increased the participation rate in clinical settings. Sending invitation letters increased the participation rate in cervical cancer screening by 1.92 percent point and 2.76 percent point (1.11+1.65) in all and urban municipalities, respectively, but the effects appear small compared to previous studies in Japan [25,26]. Free screening was verified to have the effect of increasing the participation rates in cancer screening in previous Japanese studies [22,23], but in the present analysis, it had no effect on the participation rates in screening in clinical settings for both breast and cervical cancers. Personal visits by community health workers, sending personal invitation letters and free screening are thought to be effective in improving the participation rate in mass surveys in municipalities, rather than for screening in clinical settings (S1 Table). Sending personal invitation letters was not statistically significant for the participation rate in breast cancer screening in clinical settings, but in S1 Table, this strategy had a positive effect on the participation rate in mass surveys for breast cancer. It is believed that because many municipalities had few mammography units installed in medical facilities, the residents receiving personal invitation letters could not select screening in clinical settings and would participate in mass surveys. In order to improve participation rates in breast and cervical cancer screening in clinical settings in municipalities, strategies that can improve access to cancer screening for the residents are required, because other strategies are not sufficient by themselves.

With regard to the characteristics of persons eligible for screening, similar tendencies were shown for both breast and cervical cancer screening. The percentage of 65–69 years women had a positive effect in both breast and cervical cancer screening. One possible reason is that many elderly women have a primary care physician and easy access to screening in clinical settings [49].

With regard to the characteristics of municipalities, there were some differences in the impact on the participation rates in breast and cervical cancer screening. The percentage of full-time employees among female workers had a positive effect for both breast and cervical cancer screening. In Japan, full-time employees are generally covered by health insurance provided by the workplace. Since some previous studies showed that workers with health insurance were more likely to undergo cancer screening provided in the workplace rather than that provided by a municipality, this result was unexpected [22,26–28]. The fact that full-time employees are a large fraction of female workers may not mean that there are many women who do not undergo cancer screening by municipalities, but rather that there are many women who can participate in cancer screening even during working hours. The income level of residents of a municipality increased the participation rates in screening in clinical settings only for breast cancer, which is similar to previous studies [9,22,28,29]. For cervical cancer screening in clinical settings, since there are many municipalities where the out-of-pocket costs are lower than for breast cancer, the income level probably did not affect the participation rates [50]. The percentage of persons requiring long-term care had a positive effect only for breast cancer screening. The high percentage indicates that there are many residents with a poor health status in the municipality. Since a previous study showed that women with a good self- rated health were more likely to undergo cancer screening, this result was unexpected [30]. It might be one reason why women with a poor health status had grown the awareness about prevention of disease, and so participate in cancer screening in clinical settings. The ordinary balance ratio of municipalities always had a negative effect for both breast and cervical cancer screening, while the financial capability indicator had a positive effect for cervical cancer screening. Municipalities with a low balance ratio and a high financial capability indicator have abundant financial resources, and so they can probably conduct cancer screening in clinical settings more easily than municipalities that are in a difficult financial situation [9]. In addition, because the correlation coefficient between financial capability indicators and free screening for breast and cervical cancer among municipalities were very small (0.049 and 0.148), respectively, financially abundant municipalities might make a strong effort to strategies other than free screening. The binary variable for ordinance-designated cities or the 23 wards of Tokyo had a positive effect for both breast and cervical cancer screening, while the binary variable for villages had a negative effect for breast cancer screening. It is believed that large cities have an environment in which it is easy for people to undergo screening in clinical settings, while villages do not have such an environment.

This study has several limitations that need to be addressed. First, in the analyses at the level of municipalities, the relationship between resources and cancer screening for individual residents was not evaluated. By performing the analysis at the level of individual residents, it would be possible to clarify the impact of improving access to cancer screening, such as reducing the time and distance from home to a screening facility, on the screening behavior of residents. In order to examine the impact of improving access to breast cancer screening in rural areas where the number of mammography units was very small, it might be necessary to use a different approach from that used in this study. Second, it was not possible to categorize the quantity of resources according to whether the purpose was for screening or medical care services. Even if the quantity of resources in an area is large, it is often used for medical care, so that it might not affect the participation rate in cancer screening. Third, the costs that would result from increasing the quantity of resources could not be analyzed. It is important to evaluate not only the effects, but also the cost-effectiveness, of strategies to increase participation rates in cancer screening [51,52]. There is a need to tackle these issues and further investigate the relationship between the allocation of resources and the participation rates in cancer screening in Japan.

## Conclusions

The quantity of resources for breast and cervical cancer screening affected the participation rates in screening in clinical settings in Japanese municipalities. Lack of mammography units in urban areas and of gynecologists particularly in rural areas impaired access to breast and cervical cancer screening.

Strategies are required that quickly improve access for the residents and increase their participation rates in cancer screening. For breast cancer screening, allocating more mammography units and providing mobile mammography vans should be implemented in urban areas. For cervical cancer screening, it is important to implement strategies that make screening accessible quickly, such as having Pap smears performed by primary care physicians other than gynecologists and HPV tests with self-collected samples.

## Supporting information

S1 Appendix The dataset of breast and cervical cancer screening in clinical settings in Japan in 2010. (XLSX)

S1 Fig Relationship between the participation rates in breast screening in clinical settings and the number of mammography units per 1,000 women among municipalities in Japan. (TIF)

S2 Fig Relationship between the participation rates in cervical screening in clinical settings and the number of gynecologists per 1,000 women among municipalities in Japan. (TIF)

S1 Table Determinants of the participation rates in mass surveys for breast and cervical cancer in Japan. (DOCX)
