# Supplementary figures and images for: Does lack of resources impair access to breast and cervical cancer screening in Japan?
Source: PLoS One. 2017 Jul 13;12(7):e0180819. doi: 10.1371/journal.pone.0180819 (PMC5509210; doi:10.1371/journal.pone.0180819)

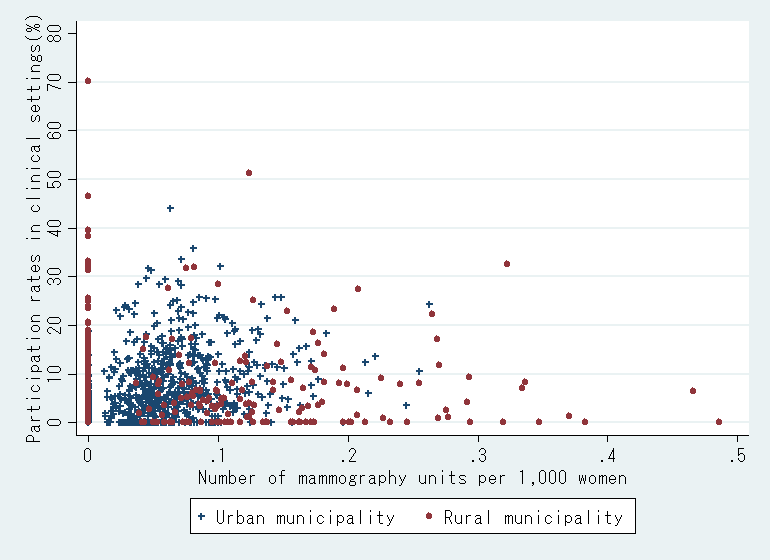

Supplement: S1 Fig — (TIF) [file pone.0180819.s002.tif]

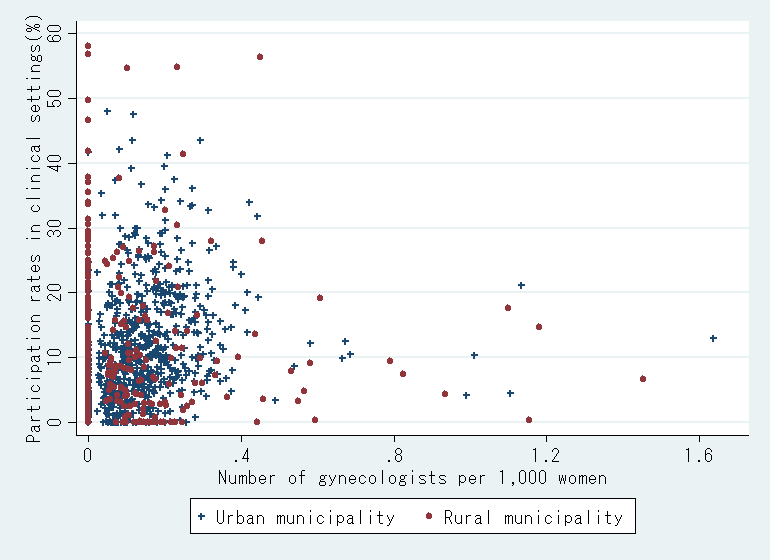

Supplement: S2 Fig — (TIF) [file pone.0180819.s003.tif]
