# Supplementary material for: Does lack of resources impair access to breast and cervical cancer screening in Japan?
Source: PLoS One. 2017 Jul 13;12(7):e0180819. doi: 10.1371/journal.pone.0180819 (PMC5509210; doi:10.1371/journal.pone.0180819)
Supplement: S1 Table — (DOCX) [file pone.0180819.s004.docx]

**S1 Table. Determinants of the participation rates in mass surveys for breast and cervical cancer in Japan.**

|  | Breast Cancer | | | | Cervical Cancer | | | |
| --- | --- | --- | --- | --- | --- | --- | --- | --- |
| Independent Variable | Coefficients | | Marginal Effects | | Coefficients | | Marginal Effects | |
| Strategies |  |  |  |  | - |  | - |  |
| Number of mammography units per 1,000 women | 2.324 |  | 2.180 |  |  |  |  |  |
|  | (5.917) |  | (5.551) |  |  |  |  |  |
| Number of mammography units per 1,000 women | -5.162 |  | -4.843 |  |  |  |  |  |
| × Urban municipality ^b)^ | (10.509) |  | (9.858) |  |  |  |  |  |
| Number of gynecologists per 1,000 women | - |  | - |  | -4.960 |  | -4.433 |  |
|  |  |  |  |  | (2.721) |  | (2.431) |  |
| Number of gynecologists per 1,000 women | - |  | - |  | 10.588 | ** | 9.461 | ** |
| × Urban municipality ^b)^ |  |  |  |  | (3.686) |  | (3.293) |  |
| Sending personal invitation letters ^b)^ | 5.796 | *** | 5.408 | *** | 5.042 | *** | 4.465 | *** |
|  | (0.784) |  | (0.727) |  | (0.683) |  | (0.599) |  |
| Sending personal invitation letters ^b)^ | -3.733 | *** | -3.456 | *** | -3.581 | *** | -3.130 | *** |
| × Urban municipality ^b)^ | (1.026) |  | (0.935) |  | (0.853) |  | (0.728) |  |
| Personal visits by community health workers ^b)^ | 3.290 | * | 3.132 | * | 4.787 | *** | 4.433 | *** |
|  | (1.422) |  | (1.370) |  | (1.242) |  | (1.182) |  |
| Personal visits by community health workers ^b)^ | -1.482 |  | -1.378 |  | -4.577 |  | -3.859 |  |
| × Urban municipality ^b)^ | (2.902) |  | (2.671) |  | (2.551) |  | (2.000) |  |
| Free screening ^b)^ | -2.717 |  | -2.506 |  | 5.641 | ** | 5.253 | ** |
|  | (2.493) |  | (2.255) |  | (1.879) |  | (1.803) |  |
| Free screening ^b)^ × Urban municipality ^b)^ | 7.448 | * | 7.199 | * | -6.784 | ** | -5.534 | ** |
|  | (3.628) |  | (3.576) |  | (2.605) |  | (1.875) |  |
| Characteristics of eligible persons |  |  |  |  |  |  |  |  |
| Number of eligible persons （1,000 women） | -0.102 | *** | -0.096 | *** | -0.022 |  | -0.020 |  |
|  | (0.030) |  | (0.028) |  | (0.017) |  | (0.015) |  |
| Percentage of those aged 65-69 years | 0.290 | *** | 0.272 | *** | 0.407 | *** | 0.364 | *** |
|  | (0.046) |  | (0.043) |  | (0.047) |  | (0.042) |  |

**S1 Table (*Continued*)**

|  | Breast Cancer | | | | Cervical Cancer | | | |
| --- | --- | --- | --- | --- | --- | --- | --- | --- |
| Independent Variable | Coefficients | | Marginal Effects | | Coefficients | | Marginal Effects | |
| Characteristics of municipalities |  |  |  |  |  |  |  |  |
| Percentage of women participating in clinical settings in 2009 | -0.271 | *** | -0.254 | *** | -0.292 | *** | -0.261 | *** |
|  | (0.011) |  | (0.010) |  | (0.010) |  | (0.009) |  |
| Percentage of female full-time employees | 0.185 | ** | 0.173 | ** | 0.201 | *** | 0.180 | *** |
|  | (0.061) |  | (0.057) |  | (0.053) |  | (0.047) |  |
| Annual income per NHI insured （1,000 yen） | -0.002 |  | -0.002 |  | 0.003 |  | 0.003 |  |
|  | (0.002) |  | (0.002) |  | (0.002) |  | (0.002) |  |
| Percentage of persons requiring long-term care | -3.146 | * | -2.952 | * | -2.945 | * | -2.632 | * |
|  | (1.586) |  | (1.488) |  | (1.374) |  | (1.228) |  |
| Ordinary balance ratio （%） | -0.121 | * | -0.114 | * | -0.069 |  | -0.061 |  |
|  | (0.050) |  | (0.047) |  | (0.046) |  | (0.041) |  |
| Financial capability indicator | 0.449 |  | 0.421 |  | 3.312 | ** | 2.960 | ** |
|  | (1.154) |  | (1.082) |  | (1.063) |  | (0.949) |  |
| Ordinance-designated city or 23 wards of Tokyo ^b)^ | 12.807 | ** | 12.514 | ** | 5.577 |  | 5.208 |  |
|  | (4.400) |  | (4.381) |  | (4.062) |  | (3.917) |  |
| Village ^b)^ | 1.172 |  | 1.105 |  | 1.850 |  | 1.677 |  |
|  | (1.112) |  | (1.054) |  | (0.970) |  | (0.892) |  |
| Constant | 23.580 | *** | - |  | 11.983 | *** | - |  |
|  | (5.414) |  |  |  | (4.837) |  |  |  |
| Number of municipalities | 1443 |  |  |  | 1469 |  |  |  |
| Number of municipalities at the participation rate = 0% | 109 |  |  |  | 219 |  |  |  |
| Pseudo R2 and likelihood-ratio Chi2 | 0.0822 |  | 926.56 | *** | 0.1183 |  | 1268.43 | *** |
| Note: *** = P values≦0.001; ** = P values≦0.01; * = P values≦0.05; Standard errors in parentheses; and b) = binary variables.  The marginal effects describe the changes in the participation rate induced by a one-unit change in each independent variable. | | | | | | | | |
